# Acute kidney injury in paediatric kidney transplant recipients

**DOI:** 10.1007/s00467-025-06655-y

**Published:** 2025-01-28

**Authors:** Barian Mohidin, Stephen D. Marks

**Affiliations:** 1 NIHR Great Ormond Street Hospital Biomedical Research Centre, University College London Great Ormond Street Institute of Child Health, London, UK; 2 Department of Paediatric Nephrology, Great Ormond Street Hospital for Children, NHS Foundation Trust, London, UK

**Keywords:** Transplant, AKI, UTI, Rejection, Recurrent disease, Obstruction

## Abstract

Acute kidney injury (AKI) in paediatric kidney transplant recipients is common. Infection including urinary tract infection (UTI) and rejection are the most common causes in children. Surgical complications often cause AKI early post-transplant, whereas BK polyomavirus nephropathy rarely occurs in the first month post-transplant. Understanding kidney physiology helps to appreciate the sensitivity of the allograft to AKI, more so than native kidneys. Although the cause of AKI is often multi-factorial, there may be an underlying process that is treatable. Eliciting the aetiology, in this regard, is of paramount importance. Pre-renal and post-renal causes of allograft dysfunction are important to distinguish from intrinsic kidney disease. Clinical information and examination of fluid balance, urine dipstick testing, blood tests, bladder and kidney transplant ultrasound, and kidney transplant biopsy remain vital assessment tools in narrowing the differential diagnosis. A careful prescribed and recreational drug history is always warranted as many drugs including supplements are nephrotoxic. Additional causes such as allograft rejection, recurrent disease, and calcineurin inhibitor toxicity need to be considered in cases of allograft dysfunction, which would not affect the native kidneys. Early detection and assessment of AKI is crucial in promoting recovery. Significant progress has been made in specific pathologies over the last 20 years, which has improved kidney allograft survival rates considerably. Research into identifying AKI biomarkers to assist early diagnosis, before the serum creatinine rises, is ongoing.

**Graphical abstract:**

**Figure:**
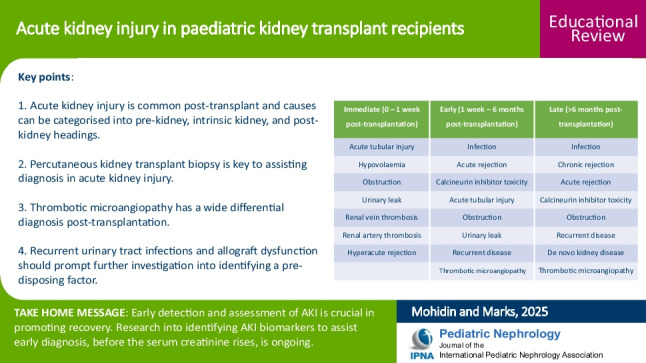
A higher resolution version of the Graphical abstract is available as Supplementary information

**Supplementary Information:**

The online version contains supplementary material available at 10.1007/s00467-025-06655-y.

## Introduction

Acute kidney injury (AKI) is defined as an increase in the serum creatinine of 26 µmol/L or greater within 48 h, or at least a 50% rise in serum creatinine known or presumed to have occurred within the past 7 days, or a fall in urine output to less than 0.5 mL/kg/h for more than 6 h [1]. There are several issues with this definition. For example, many drugs, some of which are used in transplantation, inhibit the organic cation transporter and therefore prevent tubular creatinine secretion. The trimethoprim component of co-trimoxazole is one such example. The resultant elevation in serum creatinine may fulfil the AKI criteria. However, glomerular function is unaffected which can be proven using alternative more expensive substrates which better estimate true glomerular filtration rate (GFR). On the other hand, kidney donors lose approximately 50% of their kidney function when they donate a kidney, yet their serum creatinine post-donation only slightly changes suggesting a lack of sensitivity with using creatinine as a biomarker. In other words, the relationship is non-linear and a significant decline in true GFR needs to occur before serum creatinine rises. The current definition does not capture this vulnerable group. Additionally, the paediatric population encompasses a diverse group of ages and with children having lower muscle mass and lower volumes of distribution compared to adults. This raises sensitivity issues with the current AKI definition as the same absolute rise in serum creatinine in children may reflect varying degrees of kidney dysfunction depending on the age of the child and irrespective of whether the AKI definition is met. Equally, the error margin of the creatinine assay may include a range which represents significant kidney dysfunction. On the other hand, as a child grows older and increases their muscle mass, their serum creatinine also increases proportionally, and this may represent normal physiology even if it fulfils the AKI definition. Moreover, the significance of a creatinine rise of 26 µmol/L is very different when then baseline creatinine is 70 µmol/L compared to 300 µmol/L.

More sensitive biomarkers are under investigation; however, specificity and cost remain an issue impeding widespread use [2]. Cystatin C, a low-molecular-weight protein produced by all nucleated cells, is an alternative biomarker that is gaining attraction. Research using healthy paediatric cohorts has identified age and gender-specific reference ranges [3]. However, cystatin C levels are affected by inflammation and steroids use which may limit interpretation in transplant recipients [4].

AKI in paediatric kidney transplant recipients is common with 37% of children aged between 9 and 16 years experiencing at least one episode [5]. Alkandari et al. reported the most common causes to be infection (50%), rejection (28%), and calcineurin inhibitor toxicity (11%) [5]. Urinary tract infection (UTI) contributed to one-fifth of all transplant AKIs [5]. Recurrence of AKI is also common and predisposes to chronic kidney disease in the non-transplant setting and correlates with mortality [6]. Resolution of AKI does not always return the serum creatinine back to baseline and whether AKI heralds an acceleration in the decline in graft function over time and therefore portends a worse prognosis is subject to debate [7]. Determining the cause of AKI is important as many causes are treatable. Having a structure to assess AKI is helpful as often it is multi-factorial and enables a systematic approach with pre-renal, intrinsic kidney and post-renal categories. Although AKI can occur at any time post-transplantation, some causes occur more commonly soon after transplantation whereas others predominantly occur much later. Therefore, we can also categorise causes according to time periods post-transplantation (Table 1). Other approaches are equally valid, and the emphasis is not on a particular structure, but on having a systematic approach for clinicians to undertake a comprehensive assessment.

**Table 1:** Causes of transplant acute kidney injury according to time post-transplantation

Immediate (0–1 week post-transplantation)	Early (1 week–6 months post-transplantation)	Late (> 6 months post-transplantation)
Acute tubular injury	Infection	Infection
Hypovolaemia	Acute rejection	Chronic rejection
Obstruction	Calcineurin inhibitor toxicity	Acute rejection
Urinary leak	Acute tubular injury	Calcineurin inhibitor toxicity
Renal vein thrombosis	Obstruction	Obstruction
Renal artery thrombosis	Urinary leak	Recurrent disease
Hyperacute rejection	Recurrent disease	De novo kidney disease
	Thrombotic microangiopathy	Thrombotic microangiopathy

## Pre-renal causes of acute kidney injury

Pre-renal causes are due to a disruption in the circulatory supply to the allograft. They can either be due to a problem with the pump (cardiac muscle), flow (effective circulating volume), or vascular connection (donor renal artery anastomosis). It is important to note the allograft has no sympathetic innervation as this is cut at retrieval. Consequently, autoregulation is significantly impaired, and while perturbations in blood pressure in native kidneys do not generally result in an alteration to renal plasma flow (across a tolerated range of mean arterial blood pressures), this mechanism is lacking in the allograft, and as such, less profound changes in blood pressure will significantly disrupt renal plasma flow, impairing GFR.

Cardiac output is a key component of blood pressure and is a product of heart rate and stroke volume. Cardiac failure resulting in a low cardiac output state will therefore impair flow to the allograft. The effective arterial blood volume is reduced in such a scenario resulting in impaired sodium delivery to the distal tubule, avid sodium retention through aldosterone, and further vasoconstriction in the afferent arteriole, signalled through the macula densa, further reducing GFR. This syndrome can be particularly difficult to manage in the clinical setting. It is worth noting that echocardiography is user-dependent and does not always mirror the clinical picture. The estimated ejection fraction, a useful aspect of echocardiography, can be misleading, since ejection fraction contributes little to stroke volume if end diastolic volume is also low.

An adequate and constant blood supply to the allograft is crucial for maintaining glomerular filtration and more so for the absorptive properties of tubules, particularly the segments which deal with mass nutrient reabsorption against respective concentration gradients. The relative hypoxaemic environment of the vasa recta limits oxygen supply to the proximal tubule, which houses a high density of mitochondria, which need a constant oxygen supply for aerobic respiration to form adenosine triphosphate, the key molecule which enables numerous co-transporters and exchangers to function and facilitate reabsorption of key filtered nutrients. Any disruption to blood flow therefore limits oxygen delivery, impacting ATP production and consequently tubular function. This predisposes the allograft to acute tubular injury. It is worth noting that autoregulation does not exist for tubules [8], unlike in native kidney glomeruli, and as such, allograft tubular function is particularly susceptible to changes in plasma flow, even more so than in native kidneys due to the lack of autoregulation. Equally, any process within the vasculature that limits plasma inflow to the allograft has the potential of causing AKI. Examples include hypovolaemic shock from gastrointestinal losses or haemorrhage, or re-distributive shock, where inflammatory mediators released from sepsis, anaphylaxis, or pancreatitis, triggers endothelial fluid leakage from the vascular space into the interstitial compartment.

Vascular issues are usually technical complications when they arise soon after transplantation. They may be mechanical in nature due to kinking of a long donor renal artery confined into a compact extraperitoneal space. However, most cases are due to transplant renal artery stenosis (TRAS) which can develop years after transplantation. Stenosis is most common at the anastomosis site between donor renal artery and recipient artery; however, it can occur anywhere along the arterial supply, particularly if there is an atherosclerotic burden in the recipient. Presentation is usually with allograft dysfunction, minimal proteinuria, difficult to control hypertension, and fluid overload. Hypokalaemic metabolic alkalosis is sometimes seen and suggests secondary hyperaldosteronism. Goldblatt eloquently demonstrated this in 1934 using canine models and clamping their renal arteries sequentially [9]. The reduction in perfusion to a solitary kidney (from clamping) resulted in both activation of the renin–angiotensin–aldosterone pathway and impaired excretion of salt and water, leading to volume expansion and increasing the arterial pressure [10]. Imaging modalities include Doppler kidney transplant ultrasound, CT, and MR angiography. The screening modality of choice is controversial, and each has advantages and disadvantages. However, the gold standard remains digital subtraction angiography. Treatment may include angioplasty and/or stenting, but this is not without risk, including dissection of the renal artery and embolic infarction of the allograft. Other vascular complications, including thrombotic complications, will be discussed in the next section.

## Intrinsic kidney causes of acute kidney injury

Pathology within the allograft can be limited to a single part of the nephron or affecting multiple sites. Glomerular pathology may be associated with albuminuria, whereas tubulointerstitial pathology may lead to impaired reabsorption of key molecules leading to electrolyte imbalances and metabolic complications such as osteomalacia. Vascular pathology can be secondary to systemic diseases which may be kidney-limited in phenotype such as thrombotic microangiopathy.

### Renal artery thrombosis

This is a rare but devastating complication, affecting 1.7% of paediatric kidney transplants [11]. Often, it is due to a technical issue at the anastomotic site. Surgical risk factors include kinking, torsion, or intimal injury to the renal artery. Medical risk factors include severe acute vascular rejection, recipient hypercoagulable state, or recipient hypotension. It tends to occur early after transplantation and can present with sudden pain or anuria, particularly if only one renal artery was transplanted. Management would necessitate immediate return to theatre for rescue thrombectomy; however, prognosis is poor, and often, the allograft is unsalvageable.

### Renal vein thrombosis

This complication can occur at any time, although most cases are seen soon after transplantation with an incidence of 2.3% [11]. It may be related to technical issues at the anastomosis or due to retrieval injury. Deep venous thrombosis upstream affecting the common iliac veins or inferior vena cava may extend into the donor renal vein or result in congestion, predisposing to thrombosis. Equally, a post-operative collection causing compression may be responsible. A sudden decline in urine output or disproportionate pain should warrant an urgent Doppler kidney transplant and bladder ultrasound. Reversal of arterial diastolic flow is a characteristic finding. Management warrants immediate return to theatre for emergency thrombectomy; however, the likelihood of success is limited and often the allograft is lost.

### Pseudoaneurysms and arterio-venous fistulae

Pseudoaneurysms can arise as a post-biopsy complication or secondary to persistent infections with organisms such as *Candida albicans*. They can enlarge and haemorrhage significantly leading to the development of sudden macroscopic haematuria if situated within the parenchyma. The extent of bleeding can be vast, and embolisation through interventional radiological techniques may be required. Consequent watershed infarction may occur. An arterio-venous fistula can be a rare complication of a percutaneous kidney transplant biopsy with a reported incidence of 0.1% [12]. A bruit may be present on auscultation. The malformation that occurs can enlarge and lead to significant shunting affecting perfusion. Haemorrhage is another possibility. Doppler ultrasound can be used to diagnose and monitor the extent of the arteriovenous fistula. Management is often conservative; however, if symptoms occur, then coiling or embolisation may be required.

### Pyelonephritis

UTIs are common in paediatric kidney transplant recipients. Children with congenital anomalies of the kidney and urinary tract (CAKUT) are particularly susceptible. Approximately 21% of kidney transplant recipients experience a UTI [13], and of these, 22% experience recurrence [14]. They account for one-third of hospital admissions in children [15]. Pyelonephritis is a common complication, and phenotypically may mimic allograft rejection. It is also the most common cause of bacteraemia post-transplant [16]. Recurrent UTIs are associated with worse kidney allograft survival [17, 18]. Symptoms can be subtle, and a grumbling low-level persistent C-reactive protein may be the sole abnormality. The approach to recurrent UTI is to ensure a functioning voiding system, an estimate of which can be crudely derived from pre- and post-micturition ultrasound imaging looking for post-void residual urine. It may be necessary to image the native kidneys as well as the transplant kidney to look for obstructing stones, abscesses, or a nidus for infection. Prompt removal of foreign bodies such as ureteric stents is important. A micturating cystourethrogram may help identify transplant vesico-ureteric reflux as well as stenotic segments. Nuclear imaging with a dimercaptosuccinic acid (DMSA) scan may identify consequential scarring. Treatment of recurrent UTI may benefit from a longer course of antibiotics, such as 7–10 days, to ensure adequate coverage of any occult nidus of infection [19]. However, some individuals may find additional prophylactic measures to be helpful. These include intermittent self-catheterisation, prophylactic antibiotics post-intercourse, D-mannose [20], and methenamine hippurate [21].

### BK polyomavirus nephropathy (BKPVN)

The BK virus is a polyomavirus that remains dormant in the urothelium and has the potential to reactivate in the immunosuppressed state. The Cooperative European Paediatric Renal Transplant Initiative (CERTAIN) study reported BKPVN to occur in 5% of paediatric kidney transplant recipients, while detectable viral loads in blood or plasma could be found in 33% [22]. BKPVN is associated with allograft loss [23]. The North American Pediatric Renal Trials and Collaborative Studies (NARPTCS) reported 24% of their paediatric cohort experienced allograft loss at 2 years post-BKPVN diagnosis [23]. Obstructive uropathy has been identified as an independent risk factor [24]. Classical presentation of BKPVN is asymptomatic graft dysfunction, often with a history of increased immunosuppression. Rarer presentations include transplant ureteric stenosis and haemorrhagic cystitis. The BK polyomavirus can be detected in urine and blood. It is rare for BKPVN to occur without active viraemia so quantitative PCR testing of blood or plasma for viral DNA is a useful screening and monitoring tool. Current guidelines recommend testing paediatric kidney transplant recipients monthly for the first 9 months post-transplant, and then every 3 months until 3 years post-transplant [23]. Screening is longer in children than in adults because a significant proportion of BKPVN occurs after 24 months post-transplant [22], and as such, would be missed with current adult screening protocols. If immunosuppression is increased, for example to treat rejection, resuming monthly screening for the next 3 months is advisable [23]. Viral loads of 1000–10,000 copies/mL (or equivalent) should be repeated in 2–3 weeks to assess the trend and response to interventions [23]. A percutaneous kidney biopsy is warranted if detectable BK polyomavirus in blood or plasma occurs with worsening kidney function [23]. In the context of stable kidney function, a biopsy should still be considered in patients at high immunological or virologic risk [23]. Histologically, BKPVN manifests as a tubulointerstitial nephritis (TIN) which can mimic T cell-mediated rejection (TMR). Subtle differences include the BK polyomavirus having a predilection for the medulla, intranuclear inclusion bodies, and positive SV40 viral antigen staining on immunohistochemistry (IHC). Recently, it has been reported that the JC virus (another polyoma virus) can cause allograft dysfunction and mimic BKPVN with positive SV40 IHC staining [25].

Treatment of BKPVN is reduction of the immunosuppression burden and a careful balance needs to be reached particularly when there is co-existing rejection. Ginevri et al. followed 62 children who were kidney transplant recipients for 2 years and found 21% developed BK viraemia [26]. Immunosuppression was reduced in these 13 children and all managed to clear BK viraemia after a median of 2 months without developing rejection [26]. However, Hamasaki et al. reported only a 50% BK viral clearance rate following reduction of immunosuppression in their cohort of paediatric kidney transplants recipients [27]. The aim of immunosuppression reduction is clearance of the BK viral load or a tenfold decrease at 4 weeks [23]. If this is not achieved, current guidance recommends further immunosuppression reduction [23]. Prednisolone at 5–10 mg/day may need to be added to avoid calcineurin inhibitor monotherapy [23]. We recommend testing for donor-specific antibodies (DSA) if kidney function worsens after reducing immunosuppression in patients with BKPVN and persistent BK viral loads to assist decisions on whether to re-biopsy. Other treatment options for which there is a poor evidence base include leflunomide [28], intravenous immunoglobulin (IVIg) [29], and cidofovir [30]. It remains controversial as to whether an allograft lost to BKPVN should be explanted prior to, at the time of re-transplantation, or at all, and whether complete viral clearance from the blood and urine, while of course highly desirable, are absolute prerequisites for re-transplantation [31–33]. Current paediatric guidance advises clearance of BK viral load in blood or plasma before considering re-transplantation and advises against routine allograft nephrectomy before re-transplantation, assuming BK viral load in blood or plasma is undetectable [23]. However, current guidelines admit these recommendations are derived predominantly from observational studies [23].

### Cytomegalovirus (CMV)

CMV infection has been associated with a decline in paediatric kidney transplant function [34]. In the context of immunosuppression, the recipient is exposed to CMV through reactivation of latent virus (either from the allograft or the recipient) or develops primary infection which is usually from donor-derived virus. CMV infection involves viraemia, whereas CMV disease necessitates either symptoms or evidence of tissue-invasive disease. The incidence of CMV viraemia in children after kidney transplantation is 20%, with disease in 10% [35]. Approximately 7% of children with donor-seronegative to recipient-seronegative transplants develop primary CMV infection in the first year post-transplant [36]. This is more common than in adults, perhaps due to children being more CMV naïve at the time of transplantation. The use of T cell depleting agents for induction immunosuppression increases the risk of CMV infection [37]. The highest risk is seen in the donor-seropositive to recipient-seronegative combination. Viral load can be monitored through quantitative PCR. CMV rarely causes nephritis, but colitis and hepatitis are common. Pneumonitis can be life-threatening, and colitis can be difficult to manage as the patient may not be viraemic. Therefore, colonic biopsies should be sent for CMV IHC staining regardless of viral load status if there is clinical suspicion.

Treatment of CMV infection is usually with a reduction in immunosuppression (assuming no concomitant rejection) and valganciclovir, which has better bioavailability than oral ganciclovir. Treatment of tissue-invasive disease often involves a prolonged course of intravenous ganciclovir with frequent monitoring of kidney function to guide dosing. Leukopenia is not uncommon but current guidance does not recommend changing treatment before the addition of granulocyte-colony stimulating factor or the discontinuation of other myelosuppressive medications [38]. Most transplant centres would reduce the dose of the anti-metabolite before making changes to the calcineurin inhibitor. Mammalian target of rapamycin inhibitors (mTORi) are associated with a lower incidence of CMV infection [38]. A recent multi-centre study using everolimus and low-dose ciclosporin in paediatric kidney transplant recipients had lower CMV infection and disease rates compared to standard tacrolimus and mycophenolate combinations [39]. CMV viral load should be monitored weekly during treatment [38]. The recommended duration of treatment in asymptomatic individuals is a minimum of 2 weeks and until two consecutive undetectable viral loads, if the assay is not highly sensitive [38]. CMV hyperimmune globulin can be used in severe cases although availability is limited and evidence for efficacy is lacking [40].

Resistance to valganciclovir and ganciclovir is becoming more common, particularly with the development of viral UL97 variants [41]. The incidence of resistance appears lower in children than in adults, but it is unclear whether this is due to under-reporting [42]. Resistance should be suspected in cases of treatment failure or the development of CMV viraemia during prophylaxis. Alternative agents include foscarnet, cidofovir, and maribavir, but each have troublesome side effects. ProphylaxIs with valganciclovir or a pre-emptive approach, with weekly CMV viral load monitoring and commencement of treatment once a laboratory-specified threshold is met, are recommended strategies in CMV prevention [38]. The duration of prophylaxis is controversial with most experts advising 3 months, but 6 months is recommended in high-risk groups such as donor-seropositive to recipient-seronegative combinations, those who receive T cell depleting agents, ABO-incompatible or HLA-desensitisation induction immunosuppression protocols [38]. The risk of viraemia increases on cessation of prophylaxis, and we recommend weekly surveillance for 12 weeks [38]. Following treatment of rejection with T cell depleting agents, re-initiation of prophylaxis should be considered [38]. Antiviral prophylaxis against other herpes infections should also be considered in the donor-seronegative to recipient-seronegative combination transplants [38]. This combination is also at risk of transfusion-transmitted CMV, and current guidelines advocate the use of leukoreduced or CMV-seronegative blood products [38].

### Allograft rejection

Rejection can be divided into two main categories which have been termed T cell-mediated rejection (TMR) and antibody-mediated rejection (AMR). Rejection can be further subdivided into acute or chronic, depending on the longevity of the process. Although rejection phenotypes are likely to involve both arms of the immune system, there is often one which is predominant and treatment differs according to this. With the advent of novel and more effective immunosuppression, the incidence of rejection has decreased with time; however, it still accounts for significant morbidity and graft loss, particularly in the chronic setting where therapeutic options are limited and ineffective. The incidence of acute rejection is now approximately 10% in the first 12 months post-transplant [43]. The classic textbook signs of acute rejection causing an inflamed tender kidney are seldom seen, and often, an asymptomatic rise in serum creatinine is the only abnormality. Serum creatinine is a rather insensitive biomarker of allograft injury, and research into more sensitive biomarkers is ongoing. Percutaneous kidney transplant biopsy confirms the diagnosis but has associated risks.

### T-cell-mediated rejection (TMR)

The Banff classification is used to diagnose acute and chronic TMR [44]. Acute TMR is characterised by lymphocytes infiltrating the interstitium and invading tubular epithelial cells (tubulitis) [44]. Severe cases also involve the arterial walls (arteritis), a feature which can also be seen in AMR [45]. Treatment of acute rejection is dependent on the predominant histological phenotype. Acute TMR is often treated with pulses of intravenous methylprednisolone with rabbit anti-thymocyte globulin (ATG) reserved for steroid-resistant cases or particularly severe acute TMR (Banff IIA, IIB or III) [46]. Augmenting maintenance immunosuppression is important if adherence was not an issue. This may involve the addition of corticosteroids to a corticosteroid-free regimen, increasing target trough levels of Calcineurin inhibitor (CNI) or increasing the dose of anti-metabolites [47].

Lansberg et al. reported children experience higher rates of refractory acute TMR in comparison to adults. They reported 32 of 58 children (55%) who were treated for acute TMR and then re-biopsied at a mean of 1.7 months continued to show histological changes which resembled ongoing TMR, despite creatinine levels being similar to levels observed in children with complete histological resolution. Re-treatment with pulsed steroids and/or ATG occurred in 25 of the 32 children (78%) with incomplete resolution of TMR. There was however no significant difference in graft function at 12 months irrespective of whether complete histological resolution of acute TMR had been achieved [48]. This interesting study raises a number of questions. Firstly, it again highlights the insensitivity of serum creatinine as a biomarker for monitoring response to treatment in rejection. Follow-up biopsies checking for resolution at appropriate time points may be more informative than minimally-invasive blood tests. This raises another exploratory question: What constitutes an appropriate time point? Further research is required to investigate the timescale of a complete response to treatment which leads to histological resolution. Secondly, the efficacy of steroid treatment in acute TMR is challenged given the high rates of incomplete resolution of TMR in children. In fact, no studies proposing the use of steroids in acute TMR have included histological confirmation of resolution. Thirdly, the lack of impact on graft function at 12 months argues against further burdensome treatment and questions the clinical significance of persistent inflammatory histological changes.

### Antibody-mediated rejection (AMR)

Acute AMR is characterised by evidence of acute tissue injury in the form of glomerular inflammation (glomerulitis), peritubular capillary inflammation (peritubular capillaritis), acute tubular necrosis (ATN), or acute TMA [49]. Evidence of immunological interaction and consequential tissue injury is also required for the diagnosis, so DSA detection and C4d complement deposition are key to the diagnosis [49]. However, the Banff 2019 classification recognised the imperfect sensitivity of C4d staining and specificity of pathogenic DSA, proposing alternative criteria to substitute these two conditions [50]. The alternative they proposed was the measurement of validated gene transcripts in biopsy tissue (rather than blood) that strongly associated with AMR [51]. The initial focus was on endothelial cell gene transcripts, but this moved swiftly towards molecular classifiers based on transcripts from numerous cells including immune cells involved in AMR [52, 53]. The idea this could substitute positive C4d staining or the presence of DSA is attractive; however, the costs and availability of gene transcript testing remain a major obstacle and limit use to research, and clinically, to few institutions. Such gene transcript panels and molecular classifiers have not been validated in paediatric allografts nor have they been incorporated into the Banff TMR diagnostic criteria. This represents an area for future research.

Acute AMR is often treated with pulsed intravenous methylprednisolone, plasma exchange (PLEX), IVIg, and augmentation of maintenance immunosuppression. This protocol achieved improved graft function in a case series of paediatric kidney transplant recipients [54]. Roman-Ortiz et al. experimented with eculizumab in four children with acute AMR who were refractory to the conventional treatments above. Follow-up over 32 months revealed 75% allograft survival, but two of the three remaining children had persistent DSA [55]. Kizilbash et al. reported on the use of bortezomib in 33 children with refractory acute AMR. These children had already received IVIg (90%), PLEX (78%), and rituximab (78%). Follow-up over 15 months revealed 65% allograft survival, with improvement in graft function in 36%. However, significant side effects from bortezomib were noted in 21 of the 33 children, although none were considered life-threatening [56].

AMR correlates with poor long-term kidney allograft survival, with approximately 50% of allografts being lost at 5 years [57]. Verghese et al. showed similar outcomes in children [58]. Non-adherence is a common cause of rejection in the adolescent age group, and the transition period to adult services has been identified as a particularly risky time where adherence may fall short [59]. Clinicians need to be mindful of this, and some units have adopted specialised transplant transition clinics to tackle this sensitive issue. Chronic AMR is now widely thought to be the underlying process behind the outdated term ‘chronic allograft nephropathy’, which included immunological and non-immunological causes of kidney allograft dysfunction. Antibodies to HLA class II, including anti-HLA-DQ antibodies, associate significantly with chronic AMR and allograft loss [60]. Presentation is typically progressive with a slow rise in serum creatinine, difficult to control hypertension, and heavy proteinuria. Transplant glomerulopathy and peritubular capillary basement membrane multi-layering are key hallmark biopsy findings, along with positive C4d staining and the detection of DSA in blood. Treatment options are limited other than augmenting maintenance immunosuppression. Billing et al. reported some success with IVIg and intravenous rituximab [61]. Tocilizumab has been tried in chronic AMR with variable results [62]. Cihan et al. reported on the use of ATG as salvage therapy in nine children with chronic AMR resistant to steroid, IVIG, and rituximab treatment. At 9 months, only four of the nine children had improved graft function [63]. The Transplantation Society Working Group recommended optimising immunosuppression with the re-introduction of steroids if on a steroid-free regimen and aiming for trough tacrolimus levels > 5 ng/mL as well as optimising management of cardiovascular risk factors such as hypertension, diabetes and hyperlipidaemia [64].

### Calcineurin inhibitor (CNI) toxicity

Tacrolimus and ciclosporin have revolutionised transplantation and enable excellent kidney allograft survival outcomes [65]. Their mechanism of action involves the drug binding to immunophilins; tacrolimus binds to FK binding protein-12, and ciclosporin binds to cyclophilin. This drug-immunophilin complex inhibits calcineurin, which usually dephosphorylates and thereby activates nuclear factor of activated T cells (NFAT). Once activated, NFAT translocates to the nucleus of the T cell and acts as a transcription factor to upregulate the production of pro-inflammatory cytokines, including interleukin-2, which is key for T cell activation and signal 3 of the three-signal model [66]. CNIs are metabolised by cytochrome P450 enzymes, and therefore, CNI levels can rise significantly with concurrent administration of drugs which inhibit CYP3A4. They have variable absorption in the gastrointestinal tract, and approximately 99% of absorbed tacrolimus is bound to red cells. Tacrolimus assays reflect total plasma levels, and therefore, the free unbound concentration is not measured. This can have clinical implications if aiming for a trough target because anaemia (which is very common post-transplantation) lowers the total tacrolimus levels, without necessarily affecting the free unbound drug. Increasing the dose to achieve a target trough level may increase the free unbound active drug significantly, predisposing to acute toxicity and perhaps prolonging delayed graft function. Acute toxicity tends to be dose-dependent and is related to vasoconstriction of afferent arterioles which limits glomerular filtration rate and results in ischaemic ATN. Chronic toxicity is less well understood but is evident in individuals who take CNI for other transplants or for other indications outside of transplantation and may be related to factors which promote fibrosis [67].

### Acute tubular injury/necrosis (ATN)

This is perhaps the most common finding on percutaneous kidney transplant biopsies performed within the first month after transplantation. A rise in serum creatinine is often seen, and albuminuria is typically minimal. Ischaemia–reperfusion injury is by far the most common cause particularly when cold ischaemia times are prolonged. In the immediate post-transplant setting, if an allograft stops producing urine, it is important to check the urinary catheter is not blocked, make sure the recipient is not volume depleted, and arrange urgent Doppler kidney transplant and bladder ultrasound to check vessel patency. If the above have been excluded, ATN is most likely. Other causes of ATN are important to consider and include drugs, intra-arterial contrast agents, myoglobinuria, haemoglobinuria, crystal, and cast nephropathy. With resolution of the underlying cause and enough time passing for tubular regeneration, ATN often resolves enabling the creatinine to return to baseline.

### Thrombotic microangiopathy (TMA)

There has been significant progress made in understanding the aetiology of this group of disorders in the last 20 years, as we unravel the role of complement in the immune system and license novel therapeutic agents which target specific components of the complement cascade. TMA often presents with significant kidney dysfunction, hypertension, and positive urine dipstick testing with proteinuria and haematuria. Systemic features such as microangiopathic haemolytic anaemia (MAHA) and other organ dysfunction may be present, but kidney-limited TMA is not uncommon [68]. TMA occurring post-transplant carries a wide differential diagnosis, including CNI toxicity, AMR, infection-driven, malignant hypertension, anti-phospholipid syndrome, malignancy, and recurrence of complement-mediated haemolytic uraemic syndrome (aHUS). CNI toxicity is often dose-dependent although idiosyncratic cases have also been reported [69, 70]. Management involves withdrawal of the CNI once other causes have been excluded. Belatacept use in this scenario has increased considerably and can provide a suitable alternative in low immunological risk recipients [71], who are EBV-seropositive in view of increased risk of PTLD [72]. AMR can give rise to TMA histological findings, and it can be difficult to differentiate the two. C4d staining of the peritubular capillaries is a non-specific finding of classical pathway activation of the complement cascade and can occur in both pathologies [73]. However, the location of C4d staining in the kidneys may be informative with Laskin et al. demonstrating arteriolar predominance in haematopoietic stem cell transplantation associated-TMA [74]. The presence of rising DSA may be helpful; however, the presence of DSA itself is not diagnostic as they may not be involved in complement fixation [75]. Infection-driven TMA has been reported in the literature, and viruses such as hepatitis C, CMV, and human immunodeficiency virus (HIV) have been implicated [76–78]. Management should focus on treating the underlying infection. Malignant hypertension occasionally presents with TMA and controlling blood pressure is key to limit endothelial injury [79]. Anti-phospholipid syndrome can also present with TMA and multi-organ dysfunction, and in such a scenario, may be referred to as catastrophic antiphospholipid syndrome. Anticoagulation is key, but prognosis is often poor.

Once TMA is diagnosed in a transplant, it is important to follow a pathway of investigation that includes screening for conditions such as thrombotic thrombocytopenic purpura and Shiga toxin-producing *Escherichia coli*-associated HUS. Once these entities are excluded, comprehensive genetic and acquired antibody testing for complement components and their regulators in the alternative pathway of the complement cascade can give rise to known pathogenic variants/antibodies which will help diagnose aHUS. This condition has a high risk of recurrence post-transplant, with the exception of variants in membrane co-factor protein, a membrane-bound complement regulatory protein expressed on the surface of the donor allograft [80]. The aHUS diagnosis may be made after transplant recurrence, as native kidneys may not have been biopsied, or biopsy features may be non-diagnostic showing fibrotic or hypertensive changes only. Treatment is now available in the form of intravenous eculizumab or ravilizumab, which are both C5 inhibitors, preventing the formation of the membrane attack complex, a terminal common effector pathway in the complement cascade that leads to cell injury and death. Kidney allograft survival has improved dramatically since the advent of these agents [81].

### Recurrent disease and de novo glomerulonephritis

Recurrent disease is a major cause of allograft dysfunction in both the acute and chronic settings. One such example is aHUS which has been discussed earlier. De novo glomerulonephritis affecting the allograft is also possible.

Primary focal and segmental glomerulosclerosis (FSGS) can recur rapidly and be particularly aggressive. In fact, it is the most common cause of allograft loss due to recurrent disease in children with the majority losing their allografts within 4 years of transplantation [82]. The hallmark is proteinuria, and we would recommend potential transplant recipients who are not anephric or anuric pre-transplant to have their degree of proteinuria quantified prior to transplantation to establish a baseline. Proteinuria post-transplantation usually decreases to minimal quantities over a few months. The reason for this remains unclear as this observation has been reported in the pre-CNI era, downplaying the vasoconstrictive role of CNIs on native kidneys [83]. New onset proteinuria that continues to worsen in a kidney transplant recipient who has a background of FSGS is suspicious for recurrence and should prompt a percutaneous kidney transplant biopsy (although histological changes may not be evident early on and GFR may be unaffected). Additionally, light microscopy may be unremarkable, as it takes time for sclerotic lesions to develop. Electron microscopy will reveal effacement of podocyte foot processes which may not be diffuse at this early stage. Management is controversial but current clinical practice recommendations and consensus statements include the use of PLEX and intravenous rituximab as treatment, rather than prophylaxis [84, 85]. Early initiation of PLEX has been shown to correlate with higher remission rates [86]. However, prophylactic use of PLEX and intravenous rituximab has had mixed results [87]. PLEX should be delayed by 48 h after intravenous rituximab infusion [84]. Supportive measures should be used including medications which reduce intraglomerular pressure such as angiotensin-converting enzyme inhibitors or angiotensin receptor blockers. Some experts advocate switching tacrolimus to ciclosporin, as the latter has a more potent effect on stabilising synaptopodin, a key cytoskeleton structure which supports the integrity of podocytes [88]. Early aggressive recurrence is usually a strong risk factor for further recurrence after re-transplantation [89].

Recurrence is rare in congenital nephrotic syndrome (CNS) particularly when a genetic cause has been identified. However, one-quarter of children with homozygous truncating variants in the nephrin gene (*NPHS1*) can experience a complete absence of nephrin expression. When transplanted with a kidney expressing nephrin, they can form anti-nephrin antibodies leading to a de novo glomerulonephritis manifesting with nephrotic syndrome [90, 91]. A similar phenotype has been seen in children with homozygous variants in the podocin gene (*NPHS2*) [92]. Rituximab, cyclophosphamide, and PLEX have been used to treat such patients with varying outcomes [90–92].

The pathophysiology of primary membranous glomerulonephritis (MGN) has been revolutionised recently since the discovery of numerous novel pathogenic autoantibodies. The presence of these antibodies in the recipient at transplantation confers a significant risk of recurrence, and we would recommend prophylactic immunosuppression be considered before transplantation. It would also be advisable to allow for a period of undetectable antibodies before transplantation proceeds. Intravenous rituximab has been used successfully to treat recurrent anti-phospholipase A2 receptor MGN [93] and has been incorporated into the 2021 Kidney Disease Improving Global Outcomes (KDIGO) clinical practice guidelines for the management of glomerular diseases [94].

Idiopathic membranoproliferative glomerulonephritis (MPGN), where secondary causes have been excluded, is a more common cause of kidney failure in children than in adults and carries a significant risk of recurrence and kidney allograft loss [95]. Recently, the classification of MPGN has been updated. C3 glomerulopathies including C3 glomerulonephritis and dense deposit disease carry a significant risk of recurrence post-transplant. Intravenous eculizumab has provided some success with outcomes after treatment of post-transplant recurrence [96].

Primary hyperoxaluria (PH) is an autosomal recessive metabolic disorder which has three types, of which PH type 1 is the most common, clinically relevant and severe. PH type 1 is due to a variant in the *AGXT* gene, which encodes the enzyme alanine glyoxylate aminotransferase which is synthesised by the liver. This converts glyoxylate to glycine so patients with PH type 1 accumulate glyoxylate, much of which is then converted to oxalate by hepatic lactate dehydrogenase. Oxalate is deposited in the kidneys as well as other organs causing multi-system disease including kidney stones and kidney failure. As the defect is in an enzyme synthesised by the liver, kidney transplantation does not cure the disease, and the allograft is susceptible to oxalate deposition and injury. The risk of recurrence and kidney allograft loss is therefore high. Lumasiran, a novel drug which inhibits glycolate oxidase, an enzyme which converts glycolate back to glyoxylate, reduces glyoxylate accumulation, and therefore, less is converted to oxalate. Clinical trials have shown that lumasiran reduces oxalate levels in urine giving hope this treatment can be effective in preventing recurrence post-transplantation [97]. However, longer term studies investigating kidney allograft survival are required. Nedosiran, a novel drug which inhibits hepatic lactate dehydrogenase, prevents the conversion of glyoxylate to oxalate, a common metabolic pathway for all PH types [98]. However, phase 3 clinical trials are ongoing and results awaited, but previous treatment with sequential or combined liver-kidney transplantation, which is curative for PH type 1, is unlikely to be used in the future due to the associated morbidity.

Glomerulonephritis caused by vasculitis or lupus nephritis can recur in the allograft, but this is uncommon as usual maintenance immunosuppression renders both diseases quiescent. Transplantation should be delayed until clinical quiescence is achieved for at least 6 months [99]. Antibodies may still be detectable during this period and should not prevent transplantation [99, 100]. Careful peri-operative anticoagulation planning in anti-phospholipid syndrome is crucial to reduce the risk of graft thrombosis. Biomarkers such as anti-PR3 and anti-MPO antibodies in ANCA-associated vasculitis, and complement levels and anti-dsDNA antibodies in lupus nephritis, should be monitored post-transplant, particularly if allograft dysfunction or proteinuria develops. Percutaneous kidney transplant biopsy would be definitive, and further, immunosuppression may be necessary.

IgA nephropathy (IgAN) recurs in one-third of kidney transplant recipients [101], and the presentation is usually insidious with chronic progressive kidney dysfunction, hypertension, proteinuria, and microscopic haematuria. IgAN in some adult series accounts for as much as 40% of kidney allograft loss [102]. Treatment is largely supportive and geared towards controlling hypertension and proteinuria. Sodium-glucose co-transporter type 2 inhibitors are now being used after the DAPA-CKD trial; however, transplant recipients were excluded from this study [103]. Targeted-release budesonide appears promising and has now been licensed to treat native IgAN in some European countries [104].

IgA-vasculitis (IgAV), previously known as Henoch-Schonlein purpura, is far more common in children than in adults. A nephritis with positive urine dipstick testing to blood and protein may be seen in addition to the vasculitic rash on distal extremities, gastrointestinal disturbance, and arthritis. Recurrence post-transplantation is reportedly low at 2.5% at 5 years post-transplant [105]. There does not seem to be an association with disease severity or the immunosuppression regimen used post-transplant with recurrence risk [105].

Anti-glomerular basement membrane disease (anti-GBM) rarely recurs after transplantation; however, the risk of recurrence would be high if transplantation occurred while anti-GBM antibodies were detectable in the circulation. De novo anti-GBM can also occur, particularly in recipients with Alport syndrome, where antibodies can develop to a previously unseen epitope on type IV collagen, a key component of the allograft glomerular basement membrane. These antibodies are pathogenic and can lead to kidney allograft loss [106]. The antibody may not be detectable with current commercial enzyme-linked immunosorbent assays (ELISA), as they only detect IgG antibodies to the noncollagenous-1 domain of the α3-chain of type IV collagen [107]. Western blot may be required to detect the antibody, and percutaneous kidney transplant biopsy will reveal crescentic glomerulonephritis with linear antibody staining of the glomerular basement membrane on immunofluorescence. Treatment is with PLEX and cyclophosphamide [106].

Diabetic nephropathy can recur in adult kidney transplant recipients but also after chronic exposure to CNI and corticosteroids, which are diabetogenic and can lead to post-transplant diabetes mellitus.

## Post-renal causes of acute kidney injury

Obstruction can occur anywhere along the urinary tract and may be incomplete in nature. Obstruction is not synonymous with hydronephrosis as there may be no dilatation on ultrasound in anuric patients. Hydronephrosis and hydroureteronephrosis can occur without obstruction, for example, in cases of nephrogenic diabetes insipidus. Equally, obstruction can occur without dilatation of the urinary tracts, for example, in cases of lymphoma or malignancy [108]. This can be a difficult diagnosis to make when imaging is not supportive, and diagnostic and therapeutic nephrostomy is sometimes required.

Bladder dysfunction may also contribute to obstruction particularly when there is a disruption to the autonomic nerve supply. This can be seen in diabetes and neurological conditions. Low volume, high-pressure bladders are a particular risk factor for urine to reflux back to the transplanted kidney, which pre-disposes the patient to recurrent UTI [109].

Ureteric stents are often inserted post-transplant to ensure a patent urinary drainage system. However, most urine drains outside the stents and the stents themselves can become displaced or blocked. Ischaemic strictures are not uncommon and often occur at the distal ureter or the ureterovesical anastomosis. This is likely related to the disrupted blood supply of the lower third of the ureter, which is typically derived from branches of the internal iliac artery and highlights the importance of maintaining the meso-ureter at organ retrieval and benching. Rarer causes of ureteric strictures include transitional cell carcinoma, ureteric rejection, trauma, iatrogenic disease, radiotherapy, BK polyomavirus infection, vasculitis, and systemic lupus erythematosus.

A common complication post-transplant is the development of a lymphocoele, also known as a seroma. It is due to disruption of the lymphatic vasculature at implantation and can result in the development of a fluid-filled collection, which depending on location and size can cause direct compression of the allograft, leading to obstruction, or even, compression of the transplant renal vein, resulting in thrombosis. Most lymphocoeles resolve with time and can be managed conservatively. However, if they cause issues, percutaneous drainage (often by interventional radiology) is the management of choice. Recurrence can occur which may need a more definitive surgical approach such as a peritoneal window, where fenestrations are created in the parietal layer of the peritoneum, allowing drainage of the lymphocoele internally into the peritoneum, where it is later reabsorbed.

Urine leaks are important to distinguish from lymphocoeles. They can also present as a fluid-filled collection which recurs despite draining. Leaks most commonly occur at the ureterovesical anastomosis. A fluid creatinine can be helpful to distinguish between the two, as creatinine is much higher in concentration in urine, whereas lymph fluid has a similar concentration to serum. Management of urine leaks can be challenging but are often conservative first-line with emphasis placed on urinary catheterisation to help reduce the pressure in the bladder.

## Future directions

Due to the insensitivity of serum creatinine as a biomarker for allograft impairment, novel biomarkers are under investigation to provide an alternative non-invasive way to detect allograft impairment without having to resort to invasive protocol biopsies. One promising example is donor-derived cell-free DNA (ddcfDNA), which can be detected through a blood test. The ADMIRAL study monitored ddcfDNA in approximately 1000 adult kidney transplant recipients over a 3-year period and found elevated ddcfDNA significantly associated with both subclinical and clinical allograft rejection [110]. Persistent elevation was also predictive of a 25% decline in GFR and development of DSA [110]. Dandamudi et al. studied ddcfDNA in 57 children longitudinally and found levels remained persistently elevated post-kidney transplantation, but reached a low-level steady state after 4 months, at which point serial changes became a useful tool in predicting biopsy-proven acute rejection [111]. However, similarly increased ddcfDNA levels were also found in BK viraemic children [111]. The applicability of testing young children and the potential cost impact with serial monitoring needs consideration before widespread implementation. More large-scale multi-centred studies are required before ddcfDNA can be adopted into paediatric clinical practice, and questions remain on specificity to allograft rejection, frequency of measurement, and whether it can replace protocol biopsies.

There is much interest in detecting molecular markers such as gene transcripts which are upregulated in allograft rejection. Such tests have already been incorporated into the Banff classification of AMR as an alternative to DSA or C4d criteria, although use remains limited to few specialised centres. Studies have shown selected gene expression panels to correlate well with AMR [112]. Other studies have shown AMR diagnoses enhanced when molecular classifiers were used, in addition to histology, and independent of C4d staining and DSA detection [53]. With the advancement of technology, testing of gene expression panels and molecular classifiers on formalin-fixed paraffin-embedded allograft tissue is now possible. Large multi-centre validation of these techniques is therefore possible and necessary before we can investigate whether they can replace the gold standard of histology. Validation in the paediatric population is desperately required, and extrapolation from the adult literature should not be considered sufficient.

## Key summary points

- Acute kidney injury is common post-transplant, and causes can also be categorised into pre-renal, intrinsic kidney, and post-renal headings.
- Percutaneous kidney transplant biopsy is key to assisting diagnosis in acute kidney injury.
- Thrombotic microangiopathy has a wide differential diagnosis post-transplantation.
- Recurrent urinary tract infections and allograft dysfunction should prompt further investigation into identifying a pre-disposing factor.

## Multiple-choice questions

Answers appear following the references.

1. An anuric 16-year-old young man received a kidney transplant for kidney failure of unknown diagnosis. Two months post-transplant, he developed nephrotic range proteinuria and a percutaneous kidney transplant biopsy was performed. Light microscopy and immunohistochemistry were unremarkable. Electron microscopy revealed diffuse foot process effacement. What is the most likely diagnosis?
  - Minimal change nephrotic syndrome
  - FSGS
  - Membranous glomerulopathy
  - Alport’s syndrome
  - Systemic lupus erythematosus and lupus nephritis
2. A 16-year-old young woman with systemic lupus erythematosus received a living related kidney transplant. Her serum creatinine progressively rose from 120 µmol/l to 260 µmol/l over six months. Percutaneous kidney transplant biopsy revealed features of TMA. There were no systemic features of microangiopathic haemolytic anaemia. Complement genotyping revealed a pathogenic variant in the gene encoding complement factor H. What is the most appropriate treatment?
  - Anticoagulation
  - Cyclophosphamide using the Eurolupus protocol
  - Eculizumab
  - Discontinuation of calcineurin inhibitor
  - Plasma exchange and intravenous immunoglobulin
3. A 14-year-old young man with posterior urethral valves received a living donor kidney transplant with donor blood group O and recipient blood group A. He suffered early onset vascular rejection and was treated with intravenous methylprednisolone and anti-thymocyte globulin. Two weeks post-transplant, he presented with a febrile illness with lethargy and acute graft dysfunction with a serum creatinine of 150 µmol/l. His haemoglobin had acutely dropped to 68 g/l with low haptoglobin and high reticulocyte and lactate dehydrogenase levels. The direct antiglobulin test was strongly positive for IgG and C3d. What is the most likely cause?
  - HLA class I donor specific antibodies
  - Anti-H antibodies
  - Anti-A1 antibodies
  - Anti-A2 antibodies
  - HLA class II donor specific antibodies
4. A 15-year-old EBV-seropositive young woman with a background of familial hypomagnesaemia with hypercalciuria and nephrocalcinosis received a deceased donor kidney transplant six months ago. Percutaneous kidney transplant biopsy at four months for graft dysfunction with a serum creatinine of 300 µmol/l revealed features of calcineurin toxicity so her tacrolimus was switched to belatacept. Her serum creatinine improved to 250 µmol/l in the next month, but then started to rise again. She was admitted after developing a febrile *Escherichia coli* UTI with lower urinary tract symptoms and a serum creatinine of 350 µmol/l. Which of the following is the most appropriate next step?
  - Treat her urinary tract infection
  - Switch her back to tacrolimus
  - Pulse her with intravenous methylprednisolone
  - Treat her urinary tract infection and arrange for an urgent percutaneous kidney transplant biopsy
  - Arrange for a transplant nephrectomy
5. A 14-year-old young man received a living related kidney transplant from his older sister. The mismatch was 0–0-0 at the HLA A-, B-, and DR- loci respectively. Five years later, he presented lethargic with a serum creatinine of 1000 µmol/l in the middle of the COVID-19 pandemic where he was lost to follow-up. A percutaneous kidney transplant biopsy revealed chronic antibody-mediated rejection. He admitted to stopping his immunosuppression after carefully considering the risk of contracting COVID-19 and previously being told his kidney was a perfect match. Which DSA is most likely to be detected?
  - anti-HLA A antibodies
  - anti-HLA B antibodies
  - anti-vimentin antibodies
  - anti-HLA DR antibodies
  - anti-HLA DQ antibodies

## Supplementary information

Below is the link to the electronic supplementary material.

Graphical abstract (PPTX 75.1 KB)
