# Supplementary material for: Acute kidney injury in paediatric kidney transplant recipients
Source: Pediatr Nephrol. 2025 Jan 28;40(7):2161–75. doi: 10.1007/s00467-025-06655-y (PMC12116606; doi:10.1007/s00467-025-06655-y)
Supplement: Supplementary file 1 — Graphical abstract (PPTX 75.1 KB) [file 467_2025_6655_MOESM1_ESM.pptx]

## Slide 1
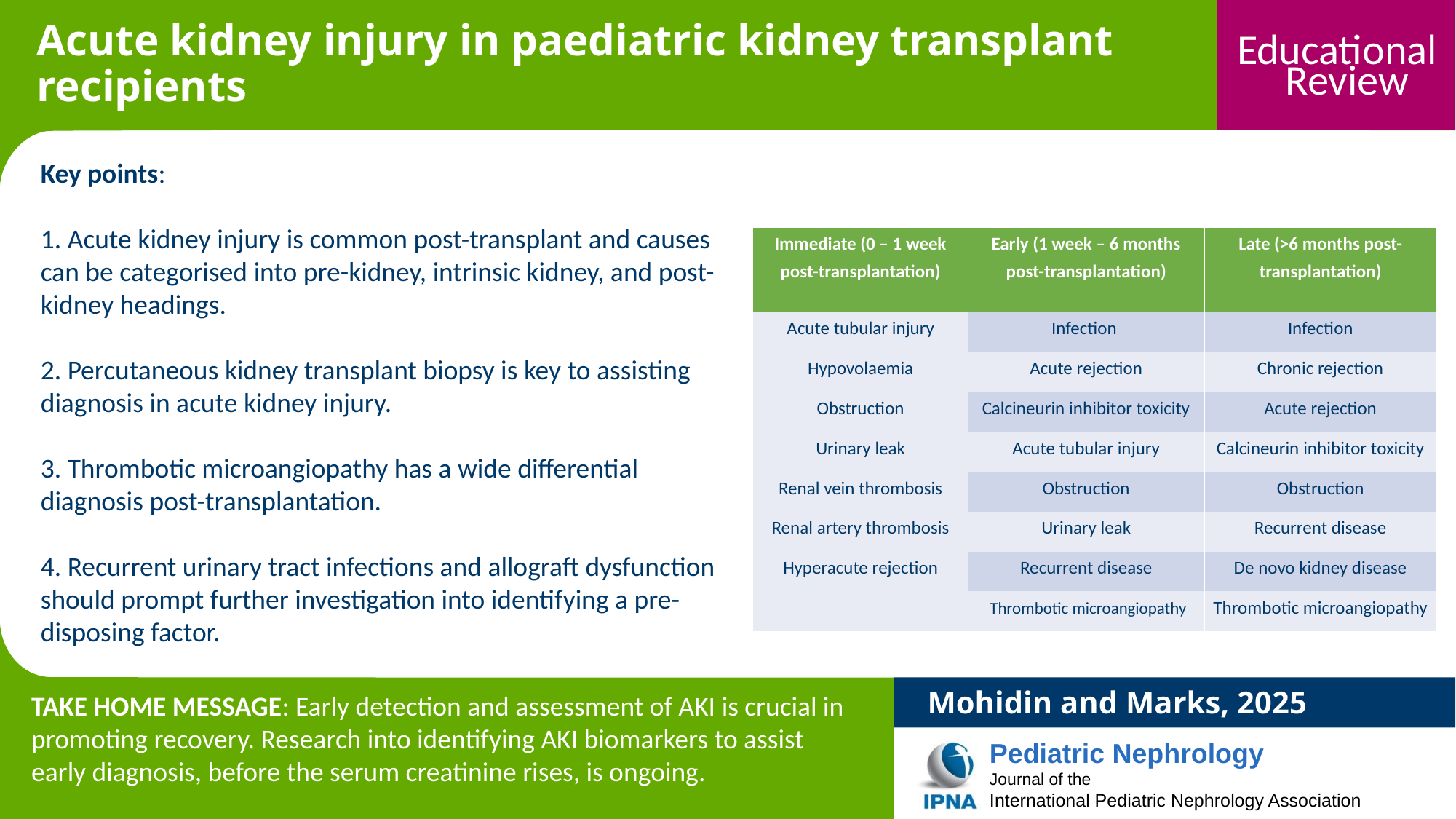

Acute kidney injury in paediatric kidney transplant recipients
Key points:
1. Acute kidney injury is common post-transplant and causes can be categorised into pre-kidney, intrinsic kidney, and post-kidney headings.
2. Percutaneous kidney transplant biopsy is key to assisting diagnosis in acute kidney injury.
3. Thrombotic microangiopathy has a wide differential diagnosis post-transplantation.
4. Recurrent urinary tract infections and allograft dysfunction should prompt further investigation into identifying a pre-disposing factor.
| Immediate (0 – 1 week post-transplantation) | Early (1 week – 6 months post-transplantation) | Late (>6 months post-transplantation) |
| --- | --- | --- |
| Acute tubular injury | Infection | Infection |
| Hypovolaemia | Acute rejection | Chronic rejection |
| Obstruction | Calcineurin inhibitor toxicity | Acute rejection |
| Urinary leak | Acute tubular injury | Calcineurin inhibitor toxicity |
| Renal vein thrombosis | Obstruction | Obstruction |
| Renal artery thrombosis | Urinary leak | Recurrent disease |
| Hyperacute rejection | Recurrent disease | De novo kidney disease |
| | Thrombotic microangiopathy | Thrombotic microangiopathy |
Mohidin and Marks, 2025
TAKE HOME MESSAGE: Early detection and assessment of AKI is crucial in promoting recovery. Research into identifying AKI biomarkers to assist early diagnosis, before the serum creatinine rises, is ongoing.
